# Genetic polymorphisms in human SULT1A1 and UGT1A1 genes associate with breast tumor characteristics: a case-series study

**DOI:** 10.1186/bcr1318

**Published:** 2005-09-21

**Authors:** Ekaterina G Shatalova, Susan E Walther, Olga O Favorova, Timothy R Rebbeck, Rebecca L Blanchard

**Affiliations:** 1 Department of Pharmacology, Fox Chase Cancer Center, Philadelphia, Pennsylvania, USA; 2 Department of Molecular Biology and Biotechnology, Russian State Medical University, Moscow, Russia; 3 Department of Fetal and Maternal Medicine, Drexel Center for Genetics, Philadelphia, Pennsylvania, USA; 4 Department of Biostatistics and Epidemiology, University of Pennsylvania School of Medicine, Philadelphia, Pennsylvania, USA; 5 Department of Clinical Pharmacology, Merck & Co., Inc., Blue Bell, Pennsylvania, USA

## Abstract

**Introduction:**

Estrogens are important in breast cancer development. SULT1A1 and UGT1A1 catalyze estrogen metabolism and are polymorphic. The SULT1A1*2 protein exhibits low activity, and a TA repeat within the UGT1A1 promoter alters the level of expression of the protein. We hypothesized that the SULT1A1*2 allozyme has decreased capacity to sulfate estrogens, that the SULT1A1*2 allele conferred increased capacity of cells to proliferate in response to estrogens, and that individuals with the variant SULT1A1 and UGT1A1 genotypes exhibited different breast tumor characteristics.

**Methods:**

The capacity for SULT1A1*2 to sulfate 17β-estradiol and the capacity for cells expressing SULT1A1*1 or SULT1A1*2 to proliferate in response to 17β-estradiol was evaluated. A case-series study was performed in a total of 210 women with incident breast cancer, including 177 Caucasians, 25 African-Americans and eight women of other ethnic background. The SULT1A1 and UGT1A1 genotypes were determined and a logistic regression model was used to analyze genotype–phenotype associations.

**Results:**

We determined that the SULT1A1*1/*1 high-activity genotype was associated with tumor size ≤2 cm (odds ratio = 2.63, 95% confidence interval = 1.25–5.56, *P *= 0.02). Individuals with low-activity UGT1A1 genotypes (UGT1A1*28/*28 or UGT1A1*28/*34) were more likely to have an age at diagnosis ≥60 years (odds ratio = 3.70, 95% confidence interval = 1.33–10.00, *P *= 0.01). Individuals with both SULT1A1 and UGT1A1 high-activity genotypes had low tumor grade (odds ratio = 2.56, 95% confidence interval = 1.04–6.25, *P *= 0.05). Upon stratification by estrogen receptor status, significant associations were observed predominantly in estrogen receptor-negative tumors.

**Conclusion:**

The data suggest that genetic variation in SULT1A1 and UGT1A1 may influence breast cancer characteristics and might be important for breast cancer prognosis.

## Introduction

**Figure 1:**
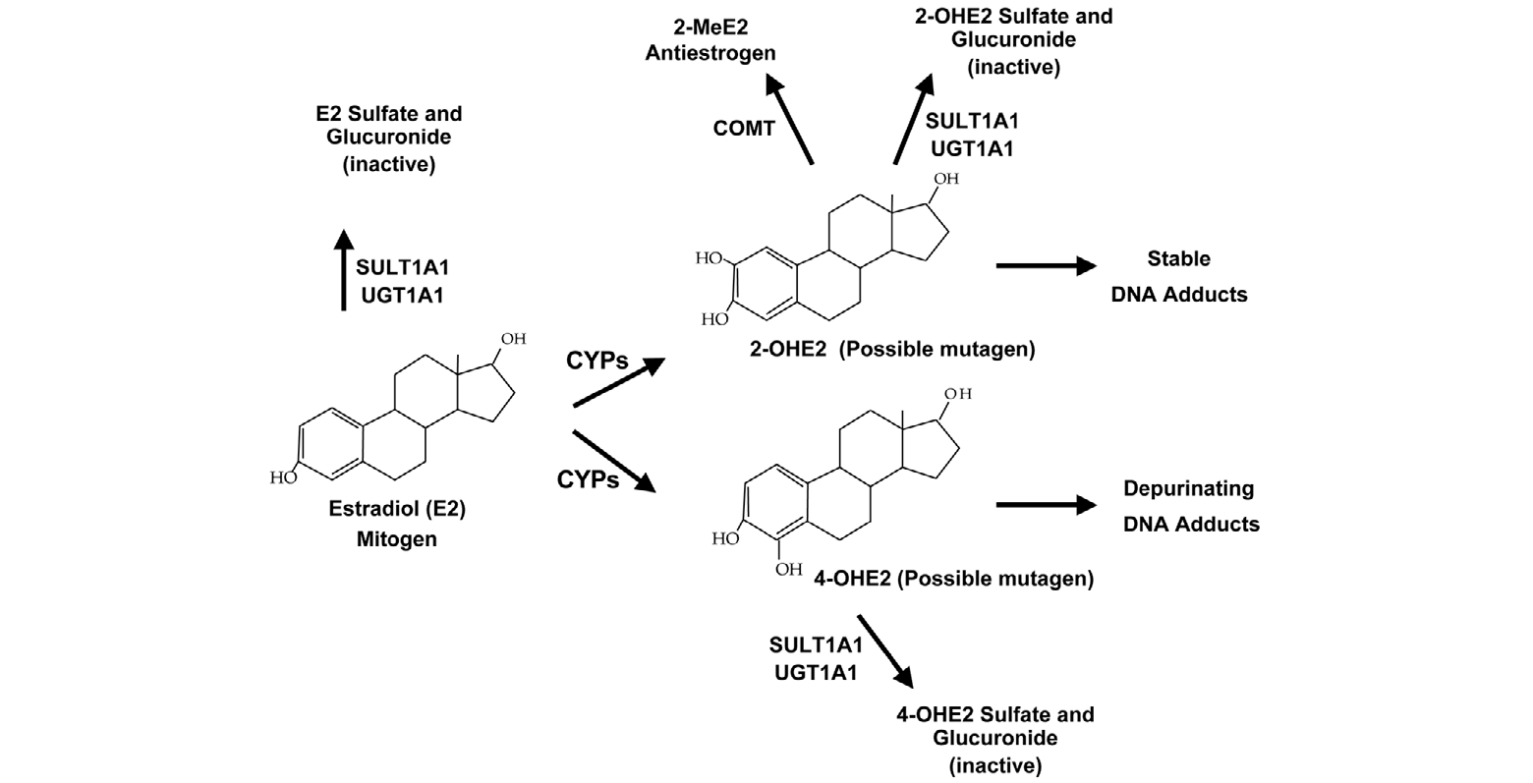
Disposition of 17β-estradiol (E2) and metabolites. SULT1A1 and UGT1A1 inactivate E2, and thus represent an antimitogenic pathway; the products of E2 oxidation, 2-hydroxyestradiol (2-OHE2) and 4-hydroxyestradiol, (4-OHE2), are both possible mutagens; 2-OHE2 is methylated by catechol-*O*-methyltransferase (COMT) to the antiproliferative compound 2-methoxyestradiol (2-MeE2), thus this pathway is potentially both antimutagenic and anitmitogenic. 2-OHE2 is alternatively sulfated and glucuronidated; this pathway would be predicted to be antimutagenic because it inactivates 2-OHE2 and, at the same time, promitogenic because it competes with the antimitogenic methylation pathway. 4-OHE2 is also inactivated by SULT1A1 and UGT1A1. Thus, sulfation and glucuronidation, depending on the cellular context and the competing metabolic pathways in a specific cell, may represent protective (detoxifying) or detrimental pathways. CYPs, Cytochromes P450.

Estrogens are involved in the development and progression of breast cancer [1-4]. Women experience various exposures to estrogens, including endogenous production, use of pharmacological estrogens (birth control medications and hormone replacement therapy) and through environmental contact. There are several mechanisms that are believed to be critical for estrogen-mediated carcinogenesis. Estrogens, being estrogen receptor (ER)-mediated mitogens, stimulate cell proliferation and promote the growth of estrogen-responsive transformed cells [5,6]. ER-independent activities of estrogens have recently been elucidated that include cell-signal transduction as well as mutagenicity [4,7-9]. Estrogens are metabolized to catecholestrogens, semiquinones and quinones, which participate in direct DNA damage and mutagenesis (Fig. 1). Those carcinogenic metabolites are the product of estrogen oxidation reactions catalyzed by the cytochrome P450 isoforms and are capable of forming either stable or depurinating DNA adducts [7,8,10], thus having the potential to result in permanent nucleotide mutation [4,9].

Conjugation of estrogens and their metabolites with methyl, sulfate or glucuronide moieties generally inactivates those molecules and is regarded as a protective reaction for the cell [4,11-13] (Fig. 1). Indeed, estrone sulfate is quantitatively the most abundant circulating hormone in humans, and deconjugation of estrone sulfate or 17β-estradiol (E2) sulfate by sulfatase is a critical reaction for liberating active estrogens within hormone-responsive tissues [4]. While conjugation of E2 and catecholestrogens is generally considered inactivating, one conjugated catecholestrogen metabolite, 2-methoxyestradiol (2-MeE2), has been recognized as a molecule with antimitogenic and antiangiogenic activities [14-16] and is now undergoing clinical study as an anticancer agent [17]. While sulfation and glucuronidation reactions may protect the cell from the mitogenic and DNA-damaging activities of E2 and catecholestrogens, those reactions also compete with methylation of 2-hydroxyestradiol (2-OHE2) and thus may decrease cellular pools of the antiproliferative 2-MeE2. Sulfation and glucuronidation, depending on cellular context and the competing metabolic pathways, may therefore represent protective (detoxifying) or detrimental (decreasing formation of 2-MeE2) pathways to the cell (Fig. 1).

Sulfotransferases and UDP-glucuronosyltransferases are two important phase II enzyme families that catalyze the sulfate and glucuronide conjugation of many endogenous and exogenous substances, including estrogens and estrogen metabolites to form water-soluble biologically inactive molecules. SULT1E1, SULT1A1 and SULT2A1 are members of a superfamily of cytosolic proteins that metabolize estrone, E2 and catecholestrogens, although the affinity of these enzymes for estrogens varies [18]. SULT1E1 has the highest affinity for estrone, E2 and catecholestrogens [11], and is expressed in many human tissues, including the liver [19] and breast, but not in malignant breast cells [20]. SULT1E1 is therefore likely to play a major role in the systemic sulfation of estrogens and catecholestrogens, and can contribute significantly to the pool of circulating estrogen sulfates [21] but perhaps not to sulfation reactions in breast tumors. SULT1A1, and to a lesser extent SULT2A1, appear to be the isoforms responsible for estrogen and catecholestrogen sulfation in breast tumors [11,18,20,22-24]. SULT1A1 also catalyzes the sulfation of 2-MeE2, an endogenous, potent antiestrogen [25]. SULT1A1 has therefore been hypothesized to be important in the sulfation of estrogens within breast tumors. In the present study we focused on SULT1A1 because this gene is polymorphic with common and well-described functionally significant alleles and is the estrogen-sulfating gene most highly expressed in breast tumors [20,22].

SULT1A1 is a polymorphic gene with three common allozymes (SULT1A1*1, SULT1A1*2 and SULT1A1*3) [26-28]. We have previously determined that the SULT1A1*2 allele, when expressed homozygously, was associated with a low level of SULT1A1 activity [26,27]. Several epidemiological studies have evaluated whether the SULT1A1 genotype is associated with altered risk for breast cancer. One study suggested a link between the high-activity SULT1A1*1 allele and early onset of breast cancer as well as the presence of non-breast tumors [29]. No evidence was found for the association of genotypes or allele frequency with tumor size, tumor stage at diagnosis and ER status [29,30]. Two additional studies have suggested a link between the low-activity allele (SULT1A1*2) and increased breast cancer risk [31,32].

UGT1A1 is a member of a superfamily of membrane-bound enzymes. Estrogenic compounds inactivated by UGT1A1 include E2, 2-hydroxyestrone, 2-OHE2, 2-MeE2 and ethinylestradiol [13,33]. UGT1A1 is expressed extensively in the liver [34] and to a lesser extent in other organs. To our knowledge UGT1A1 expression was not investigated in breast epithelium, but was detected in human breast cancer cell lines [35]. More than 60 UGT1A1 allelic variants have been described [36]. The most common UGT1A1 genetic variant is a dinucleotide TA repeat in the promoter TATA box. The wildtype allele, UGT1A1*1, contains six TA repeats, while the variant alleles UGT1A1*28, UGT1A1*33 and UGT1A1*34 contain seven, five and eight TA repeats, respectively [35,37-41]. There is an inverse relationship between the number of TA repeats at this locus and the level of transcriptional activity of UGT1A1 [35,39,42]. This polymorphism results in an altered level of enzyme expression and therefore enzymatic activity, such that UGT1A1*1 and UGT1A1*33 are associated with high activity while UGT1A1*28 and UGT1A1*34 are associated with low activity.

The UGT nomenclature has been established by the UDP Glucuronosyltransferase Nomenclature Committee. The allele containing five TA repeats in the promoter has been renamed UGT1A1*36, and the allele containing eight TA repeats has been renamed UGT1A1*37. In this manuscript we have presented our data using the previous system (UGT1A1*1, UGT1A1*28, UGT1A1*33 and UGT1A1*34) to avoid confusion when comparing our results with published literature. We have also cross-referenced the nomenclature with the historic terminology referring to the number of TA repeats.

As with SULT1A1, several studies have reported links between the UGT1A1 polymorphism and the risk of breast cancer. One study has reported a potential association between low-activity alleles (UGT1A1*28 and UGT1A1*34) and increased risk of breast cancer in premenopausal women of African ancestry (odds ratio [OR] = 1.8, 95% confidence interval [CI] = 1.0–3.1, *P *= 0.06), and the association was strongest in ER-negative breast cancer (OR = 2.1, 95% CI = 1.0–3.1, *P *= 0.04). In a larger study of Caucasian women, however, the same authors found no association between UGT1A1 polymorphisms and breast cancer [35,41]. A recent study of a Chinese population suggested that UGT1A1*28 was associated with an increased risk of breast cancer in women younger than 40 years old (OR = 1.7, 95% CI = 1.0–2.7) [43]. A reduced risk of ER-negative breast cancer was observed in a group of Caucasian and Asian patients with the UGT1A1*28/*28 genotype [44].

Given the role of SULT1A1 and UGT1A1 in the metabolism of estrogens, we hypothesized that polymorphisms in these genes might be associated with differences in breast tumor characteristics, such as tumor size, tumor grade and age of breast cancer diagnosis. Here we present data suggesting that SULT1A1*2 is associated with a low level of estrogen sulfating capacity, expression of the SULT1A1*2 allele increases the proliferative response of MCF-7 cells to estrogens and, in a case-series study of women with breast cancer, SULT1A1 and UGT1A1 alleles are significantly associated with specific tumor characteristics.

## Materials and methods

### Study subjects and data collection

A sample of 226 incident breast cancer cases (140 infiltrating ductal carcinomas, 27 infiltrating lobular carcinomas, four mixed infiltrating ductal and lobular carcinomas, one tubular carcinoma, three mucinous carcinomas, two metaplastic carcinomas and 49 unknown histological subtypes) diagnosed between 1995 and 1999 was identified through the Medical Oncology Clinics at the University of Pennsylvania Cancer Center (Table 1). Case status was confirmed by review of medical records using a standardized abstraction form. Women were excluded from this study if they were non-incident cases (i.e. those diagnosed more than 12 months prior to the date of study ascertainment) or had a prior diagnosis of cancer at any site except nonmelanoma skin cancer. The mean age of diagnosis was 59.8 years (standard deviation = 8.5 years) with a range of 43–80 years.

Information on risk factors, the medical history and breast cancer diagnostic information was obtained using a standardized questionnaire and a review of medical records. Information collected included the personal history of benign breast diseases, previous cancer diagnoses, demographic information such as race, and pathology data, including different tumor characteristics, such as the size, grade and ER status of tumors. All study subjects provided informed consent for participation in this research under a protocol approved by the Committee for Studies Involving Human Subjects at the University of Pennsylvania, and genetic samples and information were handled in accordance with the Helsinki Declaration. Genomic DNA for the present study was self-collected by each study subject using sterile cheek swabs (Cyto-Pak Cytosoft Brush; Medical Packaging Corporation, Camarillo, CA, USA).

### Generation of recombinant SULT1A1 proteins

Purified 6X-histidine-tagged recombinant SULT1A1*1 and recombinant SULT1A1*2 allozymes were generated in a baculoviral/insect cell system. SULT1A1 cDNAs were cloned into the pBlueBacHis2A expression vector (Invitrogen, Carlsbad, CA, USA). The His2A/SULT constructs were co-transfected with BacVector 3000 viral DNA (Novagen, San Diego, CA, USA) through liposome-mediated transfection into Sf-9 insect cells. After incubation at 27°C, viral supernatant was removed and diluted for isolation of individual viral clones. Clones with the highest protein expression were selected for amplification to a high-titer viral stock (>5 × 10^8 ^pfu/ml). His-tagged proteins were purified from high-titer viral stock (>5 × 10^8 ^pfu/ml) using cobalt-immobilized metal-affinity chromatography (Talon resin; Clontech, Palo Alto, CA, USA). Histidine tags were removed using the EnterokinaseMax serine protease (Invitrogen). Purified untagged recombinant SULT1A1 protein was dialyzed with 5 mM phosphate buffer (pH 6.5), and the total protein concentration was determined using the Bradford assay (Pierce, Rockford, IL, USA). Several aliquots of the purified recombinant SULT1A1 allozymes were stored at -80°C in the presence of 0.75 mg/ml BSA until assay. Purified SULT1A1 has been shown to be stable for at least 5 months under these storage conditions [45].

### SULT1A1 biochemical assay

Recombinant SULT1A1 allozymes were characterized with regard to biochemical activity toward E2 using a standard SULT1A1 radiometric assay [46,47]. One hundred nanograms of purified recombinant SULT protein was incubated with 10 μM ^35^S-labeled 3'-phosphoadenosine-5'-phosphosulfate (^35^S-PAPS), the biological sulfate donor, and varying concentrations of E2 (0–250 μM; Sigma, St. Louis, MO, USA) dissolved in dimethylsulfoxide (DMSO). The final reaction volume was 30 μl and included the standard reaction buffer containing 50 mM potassium phosphate (pH 7.4), 0.75 mg/ml BSA, 13 mM dithiothreitol and 7.5 mM MgCl_2_. The final concentration of DMSO in the reaction mixture was 3%. The reaction was initiated with the addition of 10 μM ^35^S-PAPS (NEN, Boston, MA, USA), and reactions were incubated for 15 min at 37°C and then quenched with the addition of 20 μl of a 1:1 mixture of 50 mM barium hydroxide/barium acetate. Unincorporated ^35^S-PAPS was precipitated from the reaction mixture by adding, sequentially, 10 μl of 0.1 M ZnSO_4_, 10 μl of 0.1 M (Ba)OH_2_, 10 μl of 0.1 M ZnSO_4 _and 80 μl H_2_O. The resulting precipitate was pelleted by centrifugation at 3220 × *g *for 10 min. The ^35^S-labeled reaction products were subsequently detected by liquid scintillation counting of 100 μl reaction supernatant. Each assay was performed in triplicate and 'blank samples' utilized DMSO as the vehicle control (final reaction, 3% DMSO).

The effects of substrate inhibition were largely avoided by measuring the initial reaction rates at very low substrate levels. To determine the appropriate working concentration range, a broad range of E2 concentrations were assayed initially (0–250 μM) and used to calculate initial estimates of *V*_max _(maximum reaction rate) and the Michaelis–Menten constant, *K*_m _(a measure of the affinity between substrate and enzyme measured as the substrate concentration at which half the maximal reaction rate is achieved). Assays were then repeated over a lower concentration range to fit data to the Michaelis-Menten equation to determine the apparent *V*_max _and *K*_m _values. Data were analyzed using GraphPad Prism 3.0b software (GraphPad Software, Inc., San Diego, CA, USA). Statistical significance was determined using the Mann–Whitney test.

### Generation of stably transfected MCF-7 cells

Native SULT1A1*1 and SULT1A1*2 cDNAs were cloned into the pCR3.1 expression vector (Invitrogen). MCF-7 cells were cultured in RPMI 1640 media (Cellgro, Herndon, VA, USA) and were transfected with 5 μg pCR3.1 SULT1A1*1, SULT1A1*2 or control pCR3.1 plasmids using a standard calcium phosphate method, and were cultured for 48 hours. Cells were cultured in the presence of G418 over 8 days until clones became visible to the eye. Six clones for each transfection were isolated and expanded in complete RPMI 1640 media with 10% fetal bovine serum. Clones that expressed similar levels of SULT1A1 mRNA (SULT1A1*1 or SULT1A1*2 allele) were selected for comparison of allele-dependent differences in cell proliferation.

**Figure 2:**
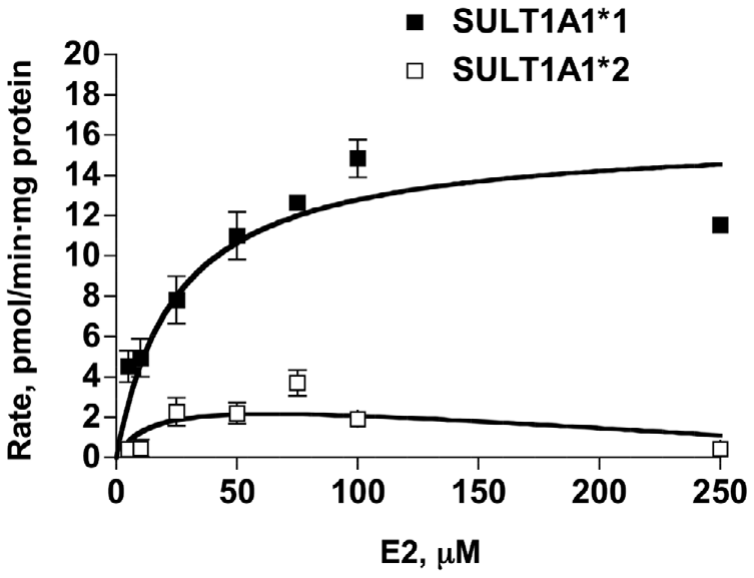
17β-estradiol (E2) sulfation by recombinant SULT1A1 allozymes. SULT1A1*2 has lower capacity to sulfate E2 than SULT1A1*1. One hundred nanograms of purified recombinant SULT1A1*1 and SULT1A1*2 allozymes were evaluated for capacity to sulfate E2 in a radiometric sulfotransferase assay. Assay conditions included 10 μM 3'-phosphoadenosine-5'-phosphosulfate (the sulfate donor) and increasing concentrations of E2 (0–250 μM). Data were evaluated on GraphPad Prism 3.0b (GraphPad Software Inc..) and fit to the Michaelis-Menten equation. SULT1A1*1, *K*_m _= 25.73 ± 7.28 μM, *V*_max _= 17.5 ± 1.71 pmol/min per mg protein; SULT1A1*2, *K*_m _= 24.74 ± 20.24 μM, *V*_max _= 3.55 ± 0.99 pmol/min per mg protein (*V*_max_, *P *< 0.0001).

We have previously determined that the biochemical mechanism by which the SULT1A1*2 allozyme is associated with low activity includes both low intrinsic turnover of substrates by the enzyme (Fig. 2) as well as faster cellular degradation of the SULT1A1*2 protein such that SULT1A1*1 has a threefold longer cellular half-life than SULT1A1*2 [48] (manuscript submitted). We have also confirmed that there is no difference in the cellular stability of the SULT1A1*1 versus SULT1A1*2 mRNA. We therefore selected clones for further proliferative analysis based on the expression of equal levels of mRNA to mimic the biological mechanisms contributing to pharmacogenetic SULT1A1 variability. Selected clones expressed equal levels of SULT1A1 mRNA, yet, as expected, western blot analysis revealed that the protein levels differed such that cells expressing SULT1A1*1 expressed threefold higher levels of SULT1A1 protein than cells expressing SULT1A1*2 (data not shown). Native MCF7 cells are heterozygous for SULT1A1*1/*2 and native levels of expression of the SULT1A1 protein are negligible compared with the levels expressed in the stable cell lines.

### Cell proliferation assay

Cell proliferation was assessed by the alamarBlue assay (BioSource International, Camarillo, CA, USA). The alamarBlue assay incorporates a water-soluble fluorometric and colorimetric indicator that is nontoxic to cells and is biotransformed to a compound detectable at 570 nm at a rate dependent upon the cell number. Cells expressing equivalent amounts of SULT1A1*1 and SULT1A1*2 mRNA and a vector-control cell line (pCR3.1) were plated in triplicate in 96-well plates at 1000 cells per well. Cells were cultured in RPMI 1640 with 10% charcoal-stripped fetal bovine serum and were washed several times over 48 hours to remove endogenous estrogens. On day 2, cells were treated with varying concentrations of E2. Proliferation was assessed as the percentage reduction of alamarBlue on day 6 by spectrophotometric absorbance at 570 nm and 600 nm on a SpectraMax Plus (Molecular Devices, Sunnyvale, CA, USA). Data were analyzed using GraphPad Prism 3.0b software (GraphPad Software). Statistical significance was evaluated using the one-way analysis of variance test (GraphPad Prism 3.0b, GraphPad Software, Inc., San Diego, CA).

### SULT1A1 genotyping assay

The SULT1A1 genotype was determined using a pyrosequencing-based assay. A 268 base pair (bp) fragment of the human SULT1A1 gene was PCR-amplified with the primers I6F395 (5'-biotin-GTTGAGGAGTTGGCTCTGCAGGGTC-3') and R733 (5'-GGGGACGGTGGTGTAGTTGGTCATAG-3'). Six nanograms of genomic DNA were mixed with TaqPlus Precision buffer (Stratagene, La Jolla, CA, USA) in a reaction mixture containing 50 mM dNTPs, 10 pmol PCR primers and 1 U TaqPlus Precision DNA polymerase in a 50 μl reaction volume. Cycling conditions included initial denaturation for 5 min at 94°C followed by 25 cycles of 94°C for 1 min, 68°C for 1 min initially with an incremental stepdown of 0.5°C on each cycle, and 72°C for 1 min. This was followed by 20 cycles of 94°C for 1 min, 56°C for 1 min and 72°C for 1 min. A final 10-min extension at 72°C completed the amplification. The presence of the PCR product was confirmed by agarose gel electrophoresis.

Amplicons were prepared for automatic pyrosequencing single nucleotide polymorphism (SNP) analysis on the PSQ 96 system (Pyrosequencing Inc., Westborough, MA, USA). Twenty-five microlitres of the double-stranded biotinylated amplicons were incubated with 100 μg streptavidin-coated DynaBeads in binding buffer (5 mM Tris, pH 7.6, 1 M NaCl, 0.5 mM ethylenediamine tetraacetic acid, 0.05% Tween 20) in a shaking thermal plate at 65°C for 15 min. Dynabeads and bound DNA were transferred to a 0.50 M NaOH solution to denature the DNA. The beads were washed and transferred to an annealing buffer (20 mM Tris acetate, pH 7.6, 5 mM Mg(OAc)_2_) for 1 min and then transferred to a sequencing primer solution containing annealing buffer and 10 pmol appropriate pyrosequencing primer.

For the G638A SNP the pyrosequencing primer was 5'-CCTCTGGCAGGGAG-3', and for the A667G SNP the primer was 5'-GAACGACGTGTGCTGAA-3'. The nucleotide dispensation sequences for the G638A and A667G SNPs were G**TC**AGCAC and ACA**TC**AGAG, respectively (underlined nucleotides representing the negative control and bold nucleotides representing the polymorphic sites). The incorporation of homozygous nucleotides generated a luciferase signal with a peak height of 2X, while heterozygous nucleotides generated peak heights of 1×. The SULT1A1 genotype was assigned as follows: SULT1A1*1, G638 A667; SULT1A1*2, A638 A667; and SULT1A1*3, G638 G667. DNA samples with known SULT1A1 genotype were also evaluated as positive control samples.

### UGT1A1 genotyping assay

UGT1A1 genotyping was performed using a PCR-based Genescan^® ^(Applied Biosystems, Foster City, CA, USA) method. A segment of the UGT1A1 gene was amplified from genomic DNA by PCR using the primers F144887 (5'-TATCTCTGAAAGTGAACTC-3') and R175122 (5'-TAGTTGTCATAGAAGGGTC-3'). These primers flank the polymorphic TA locus in the promoter region of the UGT1A1 gene, and amplify a 256 bp fragment when a (TA)_6_TAA allele is present, a 258 bp fragment when a (TA)_7_TAA allele is present, a 260 bp fragment when a (TA)_8_TAA allele is present and a 254 bp fragment when a (TA)_5_TAA allele is present. The forward primer was labeled with a fluorescent dye (6-carboxyfluorescein) at its 5'-end to permit detection of the amplified product. The amplification reaction (25 μl reaction volume) included 20 ng total human genomic DNA as template, 0.2 μM each primer, 50 μM each dNTP, 1.5 mM MgCl_2 _and 0.5 U TaqPlus Precision DNA polymerase. Reaction conditions included initial denaturation for 4 min at 95°C, followed by 35 cycles of 95°C for 30 s, 52°C for 30 s and 72°C for 30 s, followed by a final extension at 72°C for 2 min. The products were visualized by agarose gel electrophoresis.

PCR fragments were subjected to gel electrophoresis on an ABI 377 DNA analyzer (Perkin-Elmer, Wellesley, MA, USA). Amplified products were diluted in water with the addition of formamide and dextran blue loading buffer combined with a size standard (GS-350; Perkin-Elmer), were denaturated at 95°C and were loaded onto a 4% denaturing polyacrylamide gel. Fluorescent bands were analyzed using GENESCAN 2.1 software (Applied Biosystems) to determine the fragment length. Genotypes were assigned as UGT1A1*1, UGT1A1*33, UGT1A1*28, and UGT1A1*34 for six, five, seven and eight TA repeats, respectively. DNA samples with a known UGT1A1 genotype were also evaluated as positive control samples.

### Statistical methods

The chi-squared test was used to evaluate differences in allele frequencies between Caucasians and African-American subjects. Allele frequencies were not evaluated for the rest of the group (Hispanics and Asians) because of the small size of that group (11 samples; Table 1). For genotype–phenotype association analyses, genotypes were grouped based on known biological function of the alleles. SULT1A1 was grouped as follows: high activity, SULT1A1*1/*1; low activity, SULT1A1*2/*2; and intermediate or unknown function, SULT1A1*1/*2, SULT1A1*1/*3, SULT1A1*2/*3 and SULT1A1*3/*3. UGT1A1 genotype groups were grouped as: high activity, UGT1A1*1/*1, UGT1A1*1/*33 and UGT1A1*33/*33; low activity, UGT1A1*28/*28 and UGT1A1*28/*34; and intermediate or unknown function, UGT1A1*1/*28, UGT1A1*1/*34, UGT1A1*33/*28 and UGT1A1*33/*34.

We evaluated the associations between genotypes and tumor characteristics, including tumor size, tumor grade and age at diagnosis. A stepwise approach was used to identify genotype groups with a statistically significant association with tumor phenotypes. For each phenotype of interest, the chi-squared test was applied first to all genotype groups of a single gene. Genotype groups were subsequently evaluated as dichotomous variables such that high-activity genotype groups and, separately, low-activity genotype groups were tested versus all other groups.

Phenotypes were analyzed categorically. The age at diagnosis was evaluated as <60 years versus ≥60 years. This cutoff point was selected based on the mean age at diagnosis of 59.8 years with a range of 43–80 years. Tumor sizes were categorized based on those values known to be critical for prognosis and treatment strategy. Tumor size categories were ≤2 cm versus >2 cm. The tumor grade was analyzed using categories commonly applied during pathological evaluation, including grades 1, 2 or 3. Statistical analysis for the association between the genotype and the dichotomized tumor grade was performed such that grade 1 tumors were evaluated versus grade 2 and grade 3 tumors.

Logistic regression was used to estimate the ORs and 95% CIs for association of SULT1A1 and UGT1A1 genotypes with categorized tumor phenotypes, followed by a chi-squared test. ORs for age at diagnosis were adjusted for race (Caucasian versus non-Caucasian) and those for other phenotypes were adjusted for race and age at diagnosis. We used race-adjusted and age-adjusted analyses rather then race-stratified and age-stratified analyses because of the sample size limitation. Analysis with simultaneous adjustment for age at diagnosis, tumor size and grade was not performed, again because of sample size limitations. ORs, CIs and chi-squared *P *values were estimated for all subjects, among the group of subjects with ER-positive tumors and among the group of subjects with ER-negative tumors. The chi-squared test was used to estimate whether phenotype variables were distributed independently of each other. All analyses were undertaken using Minitab™ Statistical Software (Release 13.20; Minitab Inc., State College, PA, USA).

## Results

Our approach initially involved determining the functional significance of the common SULT1A1*2 allozyme toward E2 because, unlike UGT1A1, little has previously been reported regarding the functional consequences of genetic variation of SULT1A1 toward E2 biology. We then asked whether a genetic variation in UGT1A1 or SULT1A1 influenced specific tumor phenotypes. These two genes were selected for analysis because UGT1A1 and SULT1A1 directly compete for E2 and E2 metabolites as substrates.

### SULT1A1 biochemical assays

Figure 2 depicts the kinetics of those purified proteins with E2 as a substrate. The *K*_m _values for the two allozymes were similar (*K*_m _= 25.73 ± 7.28 μM and 24.74 ± 20.24 μM for SULT1A1*1 and SULT1A1*2, respectively) but the maximal rates were significantly different (*V*_max _= 17.5 ± 1.71 pmol/min per mg protein and 3.55 ± 0.99 pmol/min per mg protein for SULT1A1*1 and SULT1A1*2, respectively; *P *< 0.0001). These data clearly demonstrate that the SULT1A1*2 allozyme exhibits significantly lower capacity to catalyze the sulfation of E2. We next tested the hypothesis that MCF-7 breast carcinoma cells that were stably transfected with the SULT1A1*2 (low-activity) allele would proliferate faster in response to E2 exposure than cells expressing the SULT1A1*1 (high-activity) allele.

### Cell proliferation assay

**Figure 3:**
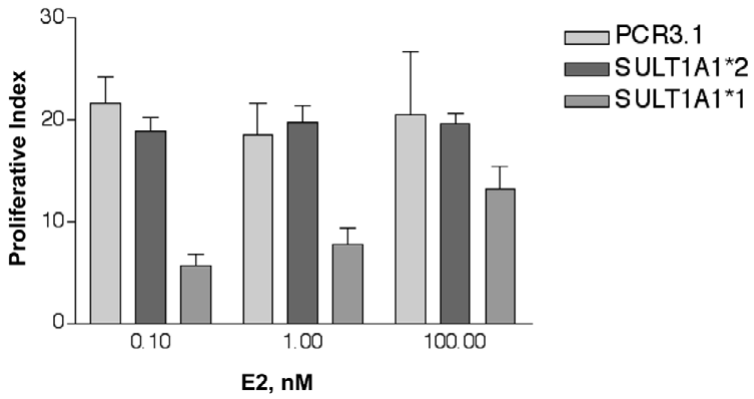
Proliferative response of MCF-7 cells overexpressing different SULT1A1 allozymes. MCF-7 cells overexpressing SULT1A1*2 proliferate faster in response to 17β-estradiol (E2) than cells overexpressing SULT1A1*1 (*P *= 0.002). Cells stably expressing equal levels of SULT1A1 mRNA (SULT1A1*1 or SULT1A1*2) were selected for study. Cells were plated onto 96-well plates and, in triplicate, treated with increasing concentrations of E2 (0, 0.1, 1 and 100 nM) in charcoal-stripped media. Data were corrected for proliferation in the absence of E2 to calculate the proliferative index. Data for 'pCR3.1' cells were generated in a MCF-7 control cell line mock-transfected with the empty pCR3.1 vector.

Figure 3 demonstrates that cells expressing SULT1A1*2 proliferated at a greater rate than cells expressing SULT1A1*1 over all three concentrations of E2 tested (*P *= 0.0022). The most significant difference in proliferation between cells expressing different SULT1A1 allozymes was observed at a physiological concentration of E2 (0.1 nM, *P *= 0.0014).

### Genotype analyses

We successfully determined the SULT1A1 and UGT1A1 genotypes from 181 subjects (80.1%) and 206 subjects (91.2%), respectively. Collectively, 210 samples of the available 226 were successfully genotyped for at least one gene. All statistical analyses were thus performed among the 210 subjects for whom we had genotype information. We did not observe differences in the distribution of characteristics presented in Table 1 between the excluded subgroup and the group that was successfully genotyped (*P *values for the chi-squared test were not statistically significant).

The allele and genotype frequencies for both genes are summarized in Table 2. The SULT1A1 genotype frequencies in Caucasian and African-American samples and the UGT1A1 genotype frequencies in Caucasians did not deviate significantly from Hardy–Weinberg proportions. SULT1A1*3 allele frequencies differed between Caucasians and African-Americans (*P *< 0.01). The UGT1A1 allele frequencies between those groups differed statistically significantly for UGT1A1*33 (*P *< 0.01), and with borderline significance for UGT1A1*1 (*P *= 0.06). The age at diagnosis was distributed independently from other phenotypes, but the tumor size and tumor grade were associated such that tumor size ≤2 cm was associated with tumor grade 1 (*P *< 0.01).

Association analyses were performed in the group of women identified as Caucasian, as well as in the entire cohort. Differences in the results were observed when non-Caucasian subjects were excluded as described in the following. Among all cases we determined that individuals with the SULT1A1*1/*1 genotype (high activity) were most likely to present with a small tumor size (≤2 cm; OR = 2.63, CI = 1.25–5.56, *P *= 0.02; Table 3) compared with individuals with other SULT1A1 genotypes. Individuals with low-activity UGT1A1 genotypes (UGT1A1*28/*28 and UGT1A1*28/*34) were more probably diagnosed at age older than 60 years (OR = 3.70, CI = 1.33–10.00, *P *= 0.01; Table 3). Individuals who carried genotypes predicting both high-activity SULT1A1*1/*1 and high-activity UGT1A1*1/*1, UGT1A1*1/*33 and UGT1A1*33/*33 presented with low tumor grade (grade 1) (OR = 2.56, CI = 1.04–6.25, *P *= 0.05; Table 3). No significant association was found between the tumor grade and the SULT1A1 or UGT1A1 genotypes when each locus was analyzed separately. In the group of women identified as Caucasian, the association between the high-activity SULT1A1 genotype and tumor size ≤2 cm (OR = 2.27, CI = 1.04–5.00, *P *= 0.02), as well as between the low-activity UGT1A1 genotypes and late age of onset (OR = 3.22, CI = 1.12–9.09, *P *= 0.02), remained statistically significant. The statistical significance of the association between the interaction of high-activity SULT1A1 and UGT1A1 genotypes with low tumor grade, however, was no longer significant within the group of only Caucasian women (OR = 1.96, CI = 0.75–5.00, *P *= 0.19).

Upon stratifying the entire group of subjects by ER status, we observed significant genotype–phenotype associations predominantly in ER-negative tumors. The SULT1A1*1/*1 high-activity genotype was associated with age at diagnosis <60 years (OR = 7.14, CI = 1.23–50, *P *= 0.02; Table 3) and tumor size ≤2 cm (OR = 14.28, CI = 2.17–100, *P *= 0.02; Table 3). The group of SULT1A1*1/*1 and UGT1A1*1/*1, UGT1A1*1/*33 and UGT1A1*33/*33 high-activity genotypes was associated with tumor size ≤2 cm (OR = 9.02, CI = 1.59–51.31, *P *= 0.01; Table 3) and low tumor grade (OR = 10, CI = 1.09–100, *P *= 0.04; Table 3). Among ER-positive tumors, we observed an association of the low-activity UGT1A1 genotype with age at diagnosis ≥60 years (OR = 9.09, CI = 1.08–100, *P *= 0.02; Table 3). In a separate analysis we compared the distribution of the SULT1A1 and UGT1A1 genotypes among women with ER-positive versus ER-negative tumors, and no difference was observed.

## Discussion

We tested the hypothesis that the SULT1A1*2 protein would exhibit impaired capacity to sulfate E2 and would confer increased proliferative response of MCF-7 cells to estrogens. The biochemical and cell culture data in Figs 2 and 3 suggest that cells exposed to physiological doses of E2 proliferated at a significantly different rate based on the SULT1A1 genotype. Approximately one-third of Caucasian and African-American individuals carry the low-activity SULT1A1*2 allele, and 10% are predicted to be homozygous for SULT1A1*2 [26,27]. Because estrogen exposure is a known factor in the etiology of breast tumors, and because the SULT1A1*2 allele is a low-activity allele with a high population frequency, we hypothesized that this allele might represent an important modifier of breast tumor characteristics. Furthermore, because a common competing metabolic pathway for SULT1A1-mediated sulfation of estrogens is UGT1A1-mediated glucuronidation of E2, we also hypothesized that UGT1A1 alleles might represent modifiers of breast tumor characteristics.

Desulfation and deglucuronidation of estrogen conjugates both contribute to the formation of active estrogen in target cells. These reactions are catalyzed by membrane-bound steroid sulfatase (arylsulfatase C) and β-glucuronidase, respectively [2,4,49]. There have been no reports of functionally significant genetic polymorphisms in those genes. We therefore did not include these genes in the current analysis. Thus, we selected SULT1A1 and UGT1A1 as candidate genes for affecting breast tumor characteristics based on the following criteria: both genes are important effectors of the biological fate of estrogens (especially pharmacological estrogens), both genes are polymorphic, and functionally significant genotype–phenotype relationships for the SULT1A1 and UGT1A1 alleles have been well characterized. Furthermore, a number of epidemiological studies have suggested a role for these genes in breast cancer risk [29,31,32,35,41,43,44], although specific results have at times been contradictory. We therefore set out to determine whether specific tumor characteristics and the age of cancer onset were influenced by the SULT1A1 or UGT1A1 alleles. To address these hypotheses we performed a case-series study of the association of the SULT1A1 and UGT1A1 genotypes with specific breast tumor characteristics, such as the size and grade of the tumor and the age at breast cancer diagnosis in a cohort of predominantly postmenopausal women with invasive breast cancer.

We observed that the high-activity SULT1A1 genotype (SULT1A1*1/*1) was associated with a greater frequency of small tumor size (≤2 cm) when compared with the group of individuals with other genotypes (Table 3). Furthermore, we observed that individuals with both high-activity SULT1A1 and high-activity UGT1A1 genotypes were more likely to have a lower tumor grade than those with other genotypes. These data are consistent with the *in vitro *data depicted in Figs 2 and 3, which clearly indicate that SULT1A1*2 is associated with a low capacity to sulfate E2 and that cells expressing SULT1A1*2 exhibit a greater proliferative response upon treatment with E2. These data are also consistent with the general notion that a high capacity to sulfate and/or glucuronidate protects the cell from the proliferative effects of estradiol. It is interesting to note that the affinity of recombinant human SULT1A1 and UGT1A1 for E2 and E2 metabolites is generally in the micromolar range. Nonetheless, the data in Fig. 3 clearly show that cells expressing high-activity SULT1A1*1 proliferate at a significantly lower rate when exposed to 1 nM E2 than cells expressing low-activity SULT1A1*2. The epidemiological analyses presented in Table 3 are consistent with these data even though it is difficult to understand from a biochemical perspective that these reactions could have such a dramatic effect on estrogenicity if endogenous levels of estrogens do not reach the micromolar range.

We also observed that the high-activity SULT1A1*1/*1 genotype had a tendency to associate with earlier age at diagnosis, although this association had borderline significance in our study. One might expect the opposite association based on the argument that a high capacity to sulfate or glucuronidate estrogens would be protective, and would therefore associate with late age of onset. However, our findings are consistent with Seth and colleagues, who also reported an association between SULT1A1*1 genotypes (homozygous or heterozygous) and early age of breast cancer onset [29]. Furthermore, we observed that low-activity UGT1A1 genotypes (UGT1A1*28/*28 and UGT1A1*28/*34) were significantly associated with later age at diagnosis. Thus, although surprising, the results reported here, as well as the data reported by Seth and colleagues [29], suggest that high-activity SULT1A1 or UGT1A1 are associated with an earlier age of onset and/or that low activity is associated with late age of onset.

Little is known about the relationship between hormones and the age of onset of breast cancer. Perhaps the explanation for this lies in the complex biology of estrogens and estrogen metabolites. For example, the estrogen metabolite 2-MeE2 is antimitogenic, and high sulfation or glucuronidation activity would be predicted to compete with methylation of 2-OHE2, resulting in lower levels of 2-MeE2. It is possible that for the phenotype of 'age of onset', the impact of conjugation on levels of 2-MeE2 is more important than the antiproliferative effects of sulfation and glucuronidation on ER-mediated proliferative activity or the antimutagenic effects of these reactions on 4-OHE2-mediated adduct formation. Such mechanistic hypotheses obviously require further studies before these questions will be answered.

The strategy of our statistical analyses was limited by the small sample size. For example, it would be interesting to adjust simultaneously for all tumor characteristics evaluated since they are not independent variables. Furthermore, we would have liked to determine the statistical significance of the differences in association between genotype and tumor characteristics in ER-negative cancers versus ER-positive cancers. Unfortunately, the small sample size, particularly for the ER-negative samples, did not allow us to have power for this analysis. Thus, although this observation was interesting, it needs to be interpreted with caution since the number of subjects for whom the ER status was known was small (Table 1). These observations might, however, generate interesting hypotheses for future testing in larger studies. Also, because of the restricted sample size we did not have enough statistical power to include all polymorphic estrogen metabolizing enzymes, such as cytochrome P450 isoforms, catechol-*O*-methyltransferase and glutathione *S*-transferase isoforms, in this analysis. Larger cohorts need to be analyzed to answer the question of interaction of all these genes and their combined effect on tumor phenotypes.

## Conclusion

The involvement of estrogens in carcinogenic processes within estrogen-responsive tissues has been recognized for a number of years [1]. Conjugation is an important biotransformation pathway for endogenous and pharmacological estrogens in humans [4]. Collectively, our *in vitro *and epidemiological data suggest that a low capacity to glucuronidate or sulfate estrogens is associated with more aggressive tumor characteristics, including larger tumor size and higher tumor grade but, curiously, later age at diagnosis.

## Abbreviations

bp = base pair; BSA = bovine serum albumin; CI = confidence interval; E2 = 17β-estradiol; ER = estrogen receptor; 2-MeE2 = 2-methoxyestradiol; 2-OHE2 = 2-hydroxyestradiol; OR = odds ratio; PCR = polymerase chain reaction; ^35^S-PAPS = ^35^S-labeled 3'-phosphoadenosine-5'-phosphosulfate.

## Competing interests

The authors declare that they have no competing interests.

## Authors' contributions

EGS contributed to the study design, carried out the genotyping, data analysis and interpretation, and drafted the manuscript. TRR was the principal investigator who directed the collection of genomic samples and epidemiological information for this cohort, participated in the study design, analysis and interpretation of the data, and critically revised the manuscript. OOF participated in the study design, data interpretation and critical manuscript revision. SEW generated the recombinant SULT1A1 proteins and stably transfected MCF-7 cells, performed the SULT1A1 biochemical assays, performed the cell proliferation assays and participated in the study design. RLB was the principal investigator who conceived of and directed the overall study, participated in the study design, interpreted data and provided critical revision of the manuscript.

## Figures and Tables

**Table 1:** Population characteristics

Characteristic	Category	Proportion (%)	Number of subjects
Race	Caucasian	84.3	177
	African-American	11.9	25
	Other	3.8	8
Age at menarche (years) (mean [standard deviation])	Known	12.7 [1.6]	208
	Unknown		3
Use of oral contraceptives	Ever	50.0	105
	Never	50.0	105
Pregnancy history	≧1 full term pregnancy	88.6	186
	No full term pregnancies	11.4	24
Age at first full term birth (years) (mean [standard deviation])	Known	24.9 [5.8]	183
	Unknown		3
Menopausal status	Premenopausal	11.9	25
	Perimenopause	5.2	11
	Postmenopausal	76.2	160
	Unknown	6.7	14
Age at menopause (years) (mean [standard deviation])		48.8 [6.2]	160
Age at diagnosis	≤60 years	62.8	132
	>60 years	34.8	73
	Unknown	2.4	5
Tumor size	≤2 cm	47.1	99
	>2 and ≤4 cm	16.7	35
	>4 cm	10.9	23
	Unknown	25.2	53
Tumor grade	1	21.4	45
	2	36.2	76
	3	23.8	50
	Unknown	18.6	39
Estrogen receptor status	Positive	42.4	89
	Negative	22.9	48
	Unknown	34.8	73

The number of subjects with each characteristic of interest is shown. The proportion of the sample size is calculated based on a total of 210 subjects that were genotyped successfully for at least one gene.

**Table 2:** SULT1A1 and UGT1A1 allele and genotype frequencies

	Caucasians^a^		African-Americans^b^	
	
	*N*^c^	Frequency	*N*^c^	Frequency
Allele				
SULT1A1*1	214	0.704	28	0.636
SULT1A1*2	87	0.286	9	0.205
SULT1A1*3	3	0.010	7	0.159
				
UGT1A1*33 (TA_5_)	5	0.015	7	0.140
UGT1A1*1 (TA_6_)	221	0.639	25	0.500
UGT1A1*28 (TA_7_)	120	0.347	13	0.260
UGT1A1*34 (TA_8_)	0	0.000	5	0.100
Genotype				
SULT1A1*1/*1	74	0.487	12	0.545
SULT1A1*1/*2	66	0.434	1	0.045
SULT1A1*1/*3	0	0.000	3	0.136
SULT1A1*2/*2	10	0.066	2	0.091
SULT1A1*2/*3	1	0.007	4	0.182
SULT1A1*3/*3	1	0.007	0	0.000
				
UGT1A1*33/*33 (TA_5_/TA_5_)	2	0.012	0	0.000
UGT1A1*33/*1 (TA_5_/TA_6_)	1	0.006	2	0.080
UGT1A1*33/*28 (TA_5_/TA_7_)	0	0.000	4	0.160
UGT1A1*33/*34 (TA_5_/TA_8_)	0	0.000	1	0.040
UGT1A1*1/*1 (TA_6_/TA_6_)	67	0.387	8	0.320
UGT1A1*1/*28 (TA_6_/TA_7_)	86	0.497	5	0.200
UGT1A1*1/*34 (TA_6_/TA_8_)	0	0.000	2	0.080
UGT1A1*28/*28 (TA_7_/TA_7_)	17	0.098	1	0.040
UGT1A1*28/*34 (TA_7_/TA_8_)	0	0.000	2	0.080

^a^Allele frequencies were defined in 152 subjects for SULT1A1 and in 173 subjects for UGT1A1.

^b^Allele frequencies were defined in 22 subjects for SULT1A1 and in 25 subjects for UGT1A1.

^c^*N*, number of alleles for allele frequency calculations or number of patients for genotype frequency calculations.

**Table 3:** Odds ratios and 95% confidence intervals for associations of SULT1A1 and UGT1A1 genotypes with breast cancer phenotypes

Phenotype		Genotype [number (%) of subjects]		Adjusted odds ratio	Adjusted 95% confidence interval	*P*
					
		High-activity SULT1A1 genotype^a^	Other SULT1A1 genotypes			
Age at diagnosis	<60 years	57 (66.28%)	48 (53.33%)	1.85^d^	1.00–3.45^d^	0.08
	≥60 years	29 (33.72%)	42 (46.67%)			
Tumor size	≤2 cm	54 (71.05%)	33(51.56%)	2.63^e^	1.25–5.56^e^	**0.02**
	>2 cm	22 (28.95%)	31 (48.44%)			
Tumor grade	1	20 (25.32%)	16 (22.86%)			
	2 or 3	59 (74.68%)	54 (77.14%)	1.36^e^	0.52–2.50^e^	0.73

		Low-activity UGT1A1 genotypes^b^	Other UGT1A1 genotypes			

Age at diagnosis	<60 years	6 (31.58%)	112 (61.88%)			
	≥60 years	13 (68.42%)	69 (38.12%)	3.70^d^	1.33–10.00^d^	**0.01**
Tumor size	≤2 cm	12 (70.59%)	86 (61.87%)	1.42^e^	0.45–4.44^e^	0.48
	>2 cm	5 (29.41%)	53 (38.13%)			
Tumor grade	1	4 (21.05%)	40 (26.85%)			
	2 or 3	15 (78.95%)	109 (73.15%)	3.70^e^	1.33–10.00^e^	0.59

		SULT1A1 and UGT1A1 high-activity genotypes^c^	Other SULT1A1 and UGT1A1 genotypes			

Age at diagnosis	<60 years	20 (57.14%)	82 (59.85%)	1.10^d^	0.52–2.35^d^	0.77
	≥60 years	15 (42.86%)	55 (40.15%)			
Tumor size	≤2 cm	19 (70.37%)	67 (59.82%)	1.79^e^	0.69–4.55^e^	0.31
	>2 cm	8 (29.63%)	45 (40.18%)			
Tumor grade	1	11 (37.93%)	24 (20.51%)	2.56^e^	1.04–6.25^e^	**0.05**
	2 or 3	18 (62.07%)	93 (79.49%)			

Associations in the group of ER-negative tumors^f^		High-activity SULT1A1 genotype^a^	Other SULT1A1 genotypes			

Age at diagnosis	<60 years	21 (91.30%)	12 (60.00%)	7.14^d^	1.23–50^d^	0.02
	≥60 years	2 (8.70%)	8 (40.00%)			
Tumor size	≤2 cm	18 (78.26%)	7 (41.18%)	14.28^e^	2.17–100^e^	0.02
	>2 cm	5 (21.74%)	10 (58.82%)			
					
		SULT1A1 and UGT1A1 high-activity genotypes^c^	Other SULT1A1 and UGT1A1 genotypes			
					
Tumor size	≤2 cm	22 (73.33%)	3 (30.00%)	9.02^e^	1.59–51.31^e^	0.01
	>2 cm	8 (26.67%)	7 (70.00%)			
Tumor grade	1	3 (37.50%)	3 (8.82%)	10.00^e^	1.09–100^e^	0.04
	2 or 3	5 (62.50%)	31 (91.18%)			

Associations in the group of ER-positive tumors^f^		Low-activity UGT1A1 genotypes^b^	Other UGT1A1 genotypes			

Age at diagnosis	<60 years	1 (14.29%)	47 (60.26%)			
	≥60 years	6 (85.71%)	31 (39.74%)	9.09^d^	1.08–100^d^	0.02

Statistically significant *P *values, as determined by the chi-squared test, are indicated in bold. ER, estrogen receptor.

^a^High-activity SULT1A1 genotype, SULT1A1*1/*1.

^b^Low-activity UGT1A1 genotypes, UGT1A1*28/*28 and UGT1A1*28/*34.

^c^High-activity UGT1A1 genotypes, UGT1A1*1/*1, UGT1A1*33/*33 and UGT1A1*1/*33.

^d^Adjusted for race (Caucasian or not Caucasian).

^e^Adjusted for age at diagnosis and race (Caucasian or not Caucasian).

^f^Only statistically significant associations are shown.
